# Supplementary material for: ΔFosB accumulation in hippocampal granule cells drives cFos pattern separation during spatial learning
Source: Nat Commun. 2022 Oct 26;13:6376. doi: 10.1038/s41467-022-33947-w (PMC9606265; doi:10.1038/s41467-022-33947-w)
Supplement: Supplementary file 1 — Supplementary Information [file 41467_2022_33947_MOESM1_ESM.pdf]

ΔFosB accumulation in hippocampal granule cells drives cFos pattern separation during spatial learning

Paul J. Lamothe-Molina, Andreas Franzelin, Lennart Beck, Dong Li, Lea Auksutat, Tim Fieblinger, Laura Laprell, Joachim Alhbeck, Christine E. Gee, Matthias Kneussel, Andreas K. Engel, Claus C. Hilgetag, Fabio Morellini and Thomas G. Oertner

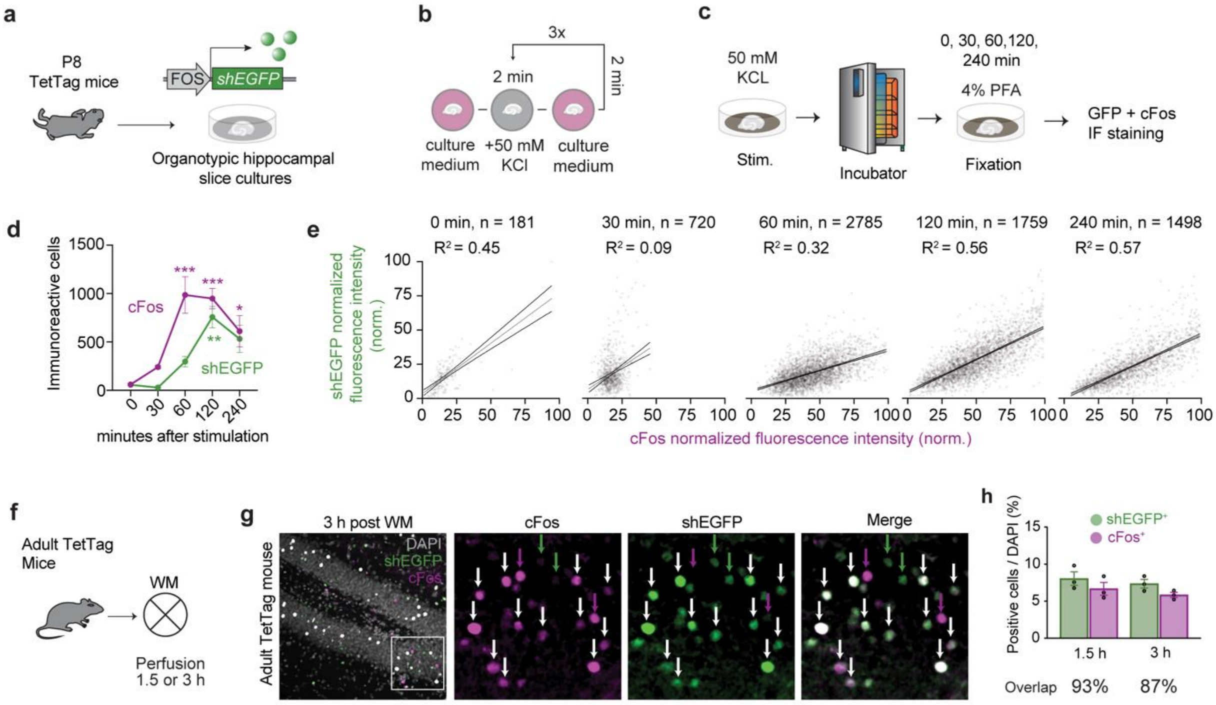

Supplementary Figure 1. Characterization of cFos reporter expression in TetTag mice. a, Hippocampal slice cultures were prepared from p8 TetTag mice in which *shEGFP* is expressed under the cFos promoter. **b**, cFos expression was induced by 50 mM potassium chloride (KCl) application. Slices were exposed to high KCl for 2 min, 3 times at 2 min intervals. **c**, Slices were returned to the incubator (37 °C) after stimulation and fixed with 4% paraformaldehyde (PFA) 0, 30, 60, 90, 120 or 240 min after the end of the stimulation. **d**, Time course of native cFos protein and shEGFP reporter expression. Immunoreactive (IR) cells were manually scored (shEGFP, n = 3 slices; cFos, n = 3 slices, mean ± SEM) inside the granule cell layer as determined by the DAPI signal. cFos$^+$ cells significantly increased after 60 min (****p < 0.0001) while shEGFP$^+$ cells significantly increased after 120 min (**p = 0.001) (two-way-ANOVA, Time Point x Protein interaction *p = 0.048. Šídák's multiple comparisons test) **e**, Anti-cFos vs. anti-shEGFP fluorescence intensity, using automatic detection of cFos+ cells (low detection threshold). High linear correlation was found 120 min and 240 min after stimulation. **f,** TetTag mice were trained in the WM to induce cFos and sacrificed 1.5 h or 3 h after the end of the last training session. **g,** Dentate gyrus from TetTag mouse stained against cFos (magenta) and shEGFP (green) 3 h after water maze (WM) training. Most fluorescent granule cells (GCs) are double-positive (white arrows). Magenta and green arrows correspond to cFos$^+$ or shEGFP$^+$ GCs, respectively. **h**, Fraction of positive cells and overlap of shEGFP reporter (green) and cFos protein (magenta) in animals sacrificed 1.5 h (n = 3 mice) and 3 h (n = 3 mice) after water maze training. Symbols correspond to individual animals, bars show mean +/- SEM.

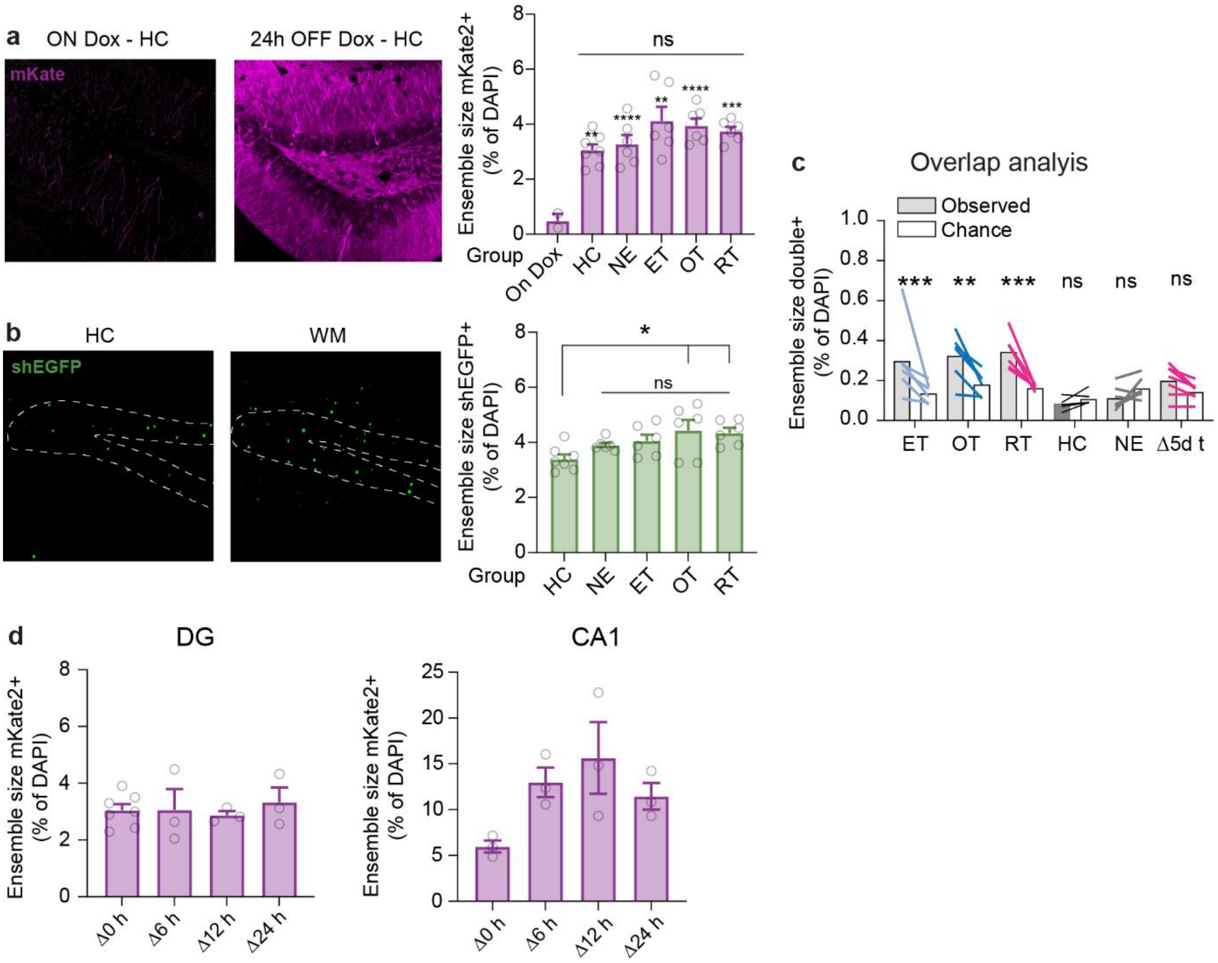

Supplementary Figure 2. Size of cFos ensembles in TetTag mice. a, Removal of Doxycline food induced cFos-dependent expression (cFos-tagged) of membrane-targeted mKate2 in the home cage (HC). The fraction of mKate2-positive neurons was not different in home-caged animals compared to novel environment (NE) and mice trained in the water maze (early training ET; overtrained OT; reversal training RT, mean +/- SEM). All groups showed significantly larger cFos^{+} ensemble sizes than mice on Dox (HC **, n = 7; NE ****, n = 6; ET ***, n = 6; OT ****, n = 6; RT ***, n = 6. One-way-ANOVA, Sidak multiple comparison test). **b**, Nuclear shEGFP reports cFos-expression in the 2-3 hours before sacrifice. Ensemble size (mean +/- SEM) was significantly larger in the OT and RT groups compared to the HC group. Ensemble size was similar in all WM-trained groups (HC vs. OT *, n = 6; HC vs. RT *, n = 6; HC vs. NE ns, n = 6; HC vs. ET ns, n = 6. One-way-ANOVA, Sidak multiple comparison test). **c**, Observed (grey) and chance-level (white) overlap between mKate2 and shEGFP-tagged ensembles from different (Figs. 1, 2 and 5) experimental groups (HC ns, n = 7 (two sets of lines are overlapping); NE ns, n = 6; ET ***, n = 6; OT **, n = 6; RT ***, n = 6; Δ5d ns. Two-way-ANOVA, Sidak's multiple comparison test). **d**, Ensemble size (mKate2, mean +/- SEM) in home caged mice sacrificed at different times after the end of a 24 h off-Dox period (see Fig. 5). Note delayed increase of mKate2-positive pyramidal cells in CA1.

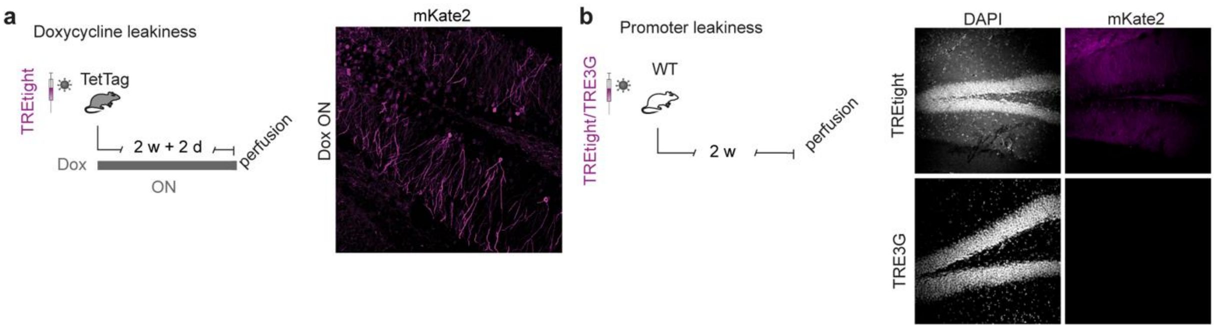

Supplementary Figure 3. Optimization of cFos tagging in TetTag mice. **a**, Doxycycline leakiness test. Mice were always ON Dox. They were injected with AAV9-TREtight-*iChloC-mKate2* bilaterally in DG and were always in their home cage (HC). Few mKate2-expressing granule cells (GCs) could be detected. **b**, Promoter leakiness test. Wild-type mice that do not express the tetracycline-transactivator (tTA) were injected with either TREtight or TRE3G-promoter region constructs using AAVs. Right: mKate2 expression was observed in TREtight, but not with TRE3G promoter.

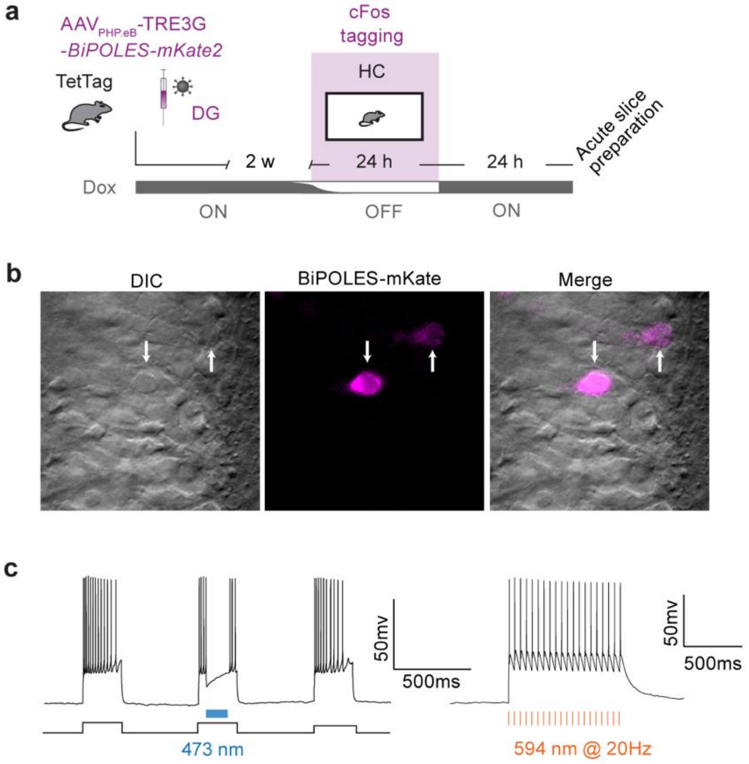

Supplementary Figure 4. BiPOLES characterization in granule cells (GCs). **a**, TetTag mice were injected with AAVPHP.eB-TRE3G-*BiPOLES-mKate2*. Mice were taken off Dox while they were in their home cage (HC). 24 h later, acute slices were prepared. **b,** Representative images showing the GC layer and BiPOLES-expressing cells. Left differential interference contrast (DIC) image, middle: fluorescent image showing mKate2 (arrows) in the soma of GCs, right: overlay. **c,** Whole cell patch-clamp recordings from BiPOLES-expressing GCs. 473 nm light prevents action potentials (APs) induced by somatic 150 pA (500 ms) current injection. 594 nm light pulses reliably elicit APs.

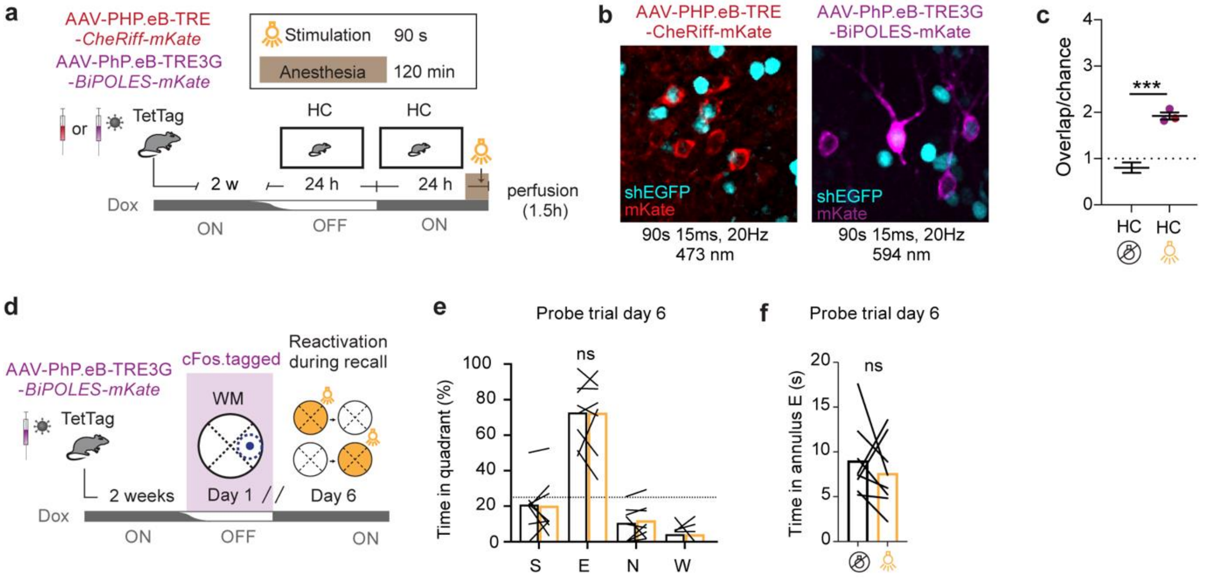

Supplementary Figure 5. Effect of optogenetic reactivation of cFos-tagged GCs on spatial memory recall. a, Testing the effect of optogenetic depolarization in home-caged (HC) mice. Mice were taken off Dox for 24 h to express the optogenetic construct in $cFos^{+}$ cells. Two different channelrhodopsins were tested, CheRiff(red) and Chrimson (magenta), the depolarizing actuator in the BiPOLES construct. Animals were light-stimulated under anesthesia (473 nm for CheRiff, 594 for BiPOLES) and sacrificed 1.5 h later. **b, c**, Light stimulation increased the overlap between the first set of $cFos^{+}$ neurons (red/magenta) and shEGFP expression (cyan). Lines show mean +/- SEM. The difference to unstimulated mice expressing mKate (HC group from Fig. 2) was significant (***p = 0.0003, two-sided, unpaired t test). **d**, Testing the effect of optogenetic reactivation of the cFos ensemble tagged on day 1 of WM training. All mice had two probe trials on day 6, one of which was performed under reactivation conditions (594 nm light pulses at 20 Hz). **e**, On day 6, performance in probe trials with optogenetic reactivation (orange bars) was not different from trials without reactivation (black bars) (ns, p > 0.99, matched two-way-ANOVA with Šídák's multiple comparisons test. **f**, Time spent in target (E) annulus was also not affected in trials with optogenetic reactivation (orange bar) (ns, p = 0.49, two-sided paired t test).

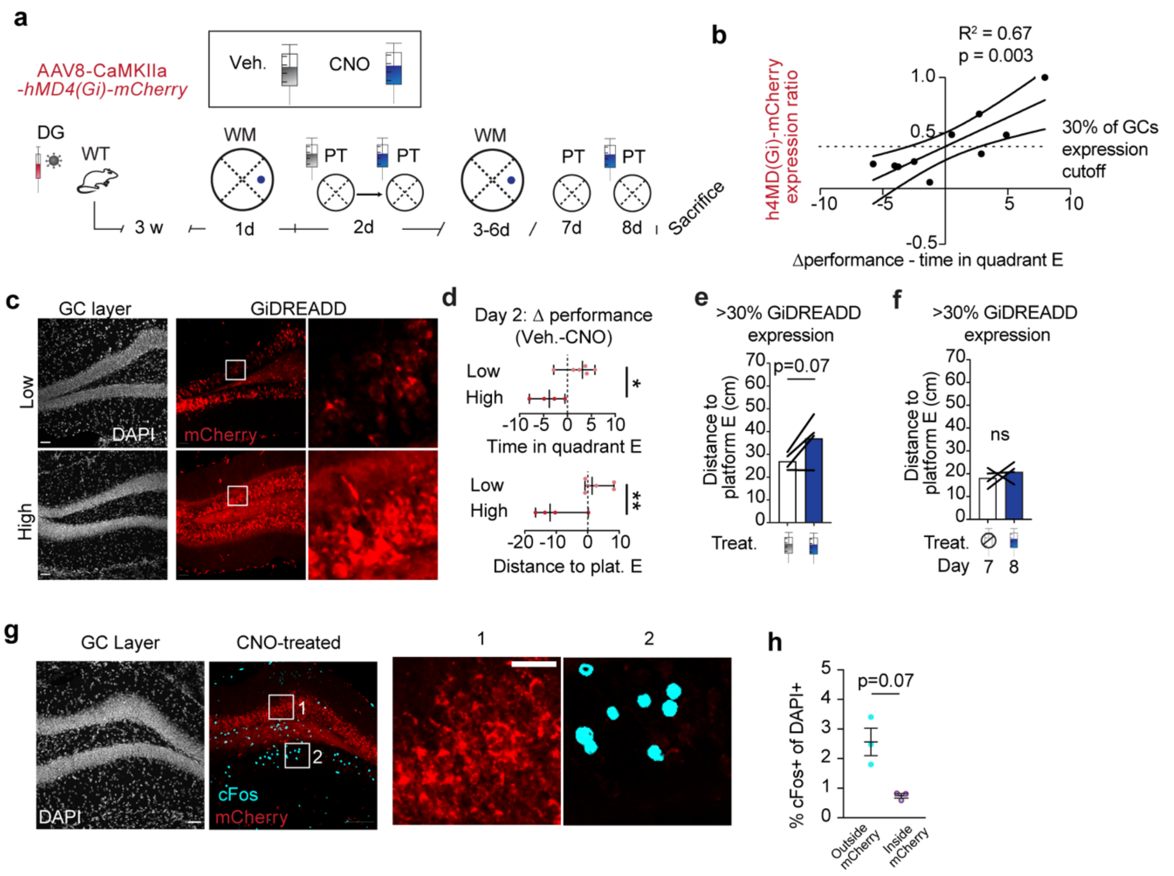

Supplementary Figure 6. Chemogenetic silencing of dentate gyrus affects spatial memory recall. a, Experimental design. Wild-type (WT) mice were injected with AAV8-CaMKIIa-*hMD4(Gi)-mCherry* bilaterally in DG (n = 10 mice). 3 weeks after injection, mice were trained in the water maze (WM) on day 1. On day 2, mice were tested for reference memory with two probe trials (PT, no platform). All mice were injected with 0.9% NaCl (vehicle) before the first PT and then with clozapine-N-oxide (CNO, 5 mg/kg) before the second PT. Both injections were done intraperitoneally (i.p.) 40 min before each PT. Mice were trained further and tested again on day 7 for reference memory at the end of that session (no treatment) and on the following day (day 8) 40 min after CNO injection (without further training). Mice were sacrificed in different batches on different days with/without CNO application for immunohistochemistry. **b**, Expression of chemogenetic silencing receptors (hMD4(Gi)-mCherry) in DG strongly correlates with performance difference (with/without CNO) on day 2. For each mouse, we calculated the difference of time spent in quadrant E after CNO injection vs vehicle injection (Δ time) as a performance measure, with positive values indicating CNO-induced decrease in performance. **c**, Representative confocal images showing hMD4(Gi)-mCherry expression in DG. Upper row: low Gi-DREADD-expressing mouse; lower row: high Gi-DREADD-expressing mouse. Left, DAPI. Middle, mCherry. Right, magnification from GCs expressing mCherry in the upper blade of the DG. Mice were separated into two groups based on their mCherry expression ratio: high > 0.5 (n = 4), low < 0.5 (n = 6). **d**, Mice in the low expression group are unaffected or show better performance on the second PT, while mice in the high expression group perform worse (Markers: individual mice; Lines: median ± min/max; Distance to platform E: *p = 0.01; Time in quadrant E: **p = 0.007; two-sided unpaired t-test). **e**, Performance was worse during chemogenetic silencing in the high expression group, however, not statistically significant (ns p = 0.07, two-sided paired t-test). **f**, Spatial memory recall is not affected in expert mice. **g**, Example from a mouse where only the upper blade of DG was transduced with Gi-DREADD (red). No cFos expression (cyan) in granule cells of the upper blade (1), but strong cFos expression in GCs of the lower blade (2). This mouse received CNO before a PT and was sacrificed 1.5 h after. **h**, Data from three mice with uneven Gi-DREADD expression, comparing cFos expression outside and inside the (mCherry-labeled) Gi-DREADD expression area (mean +/- SEM, two-sided paired t test).

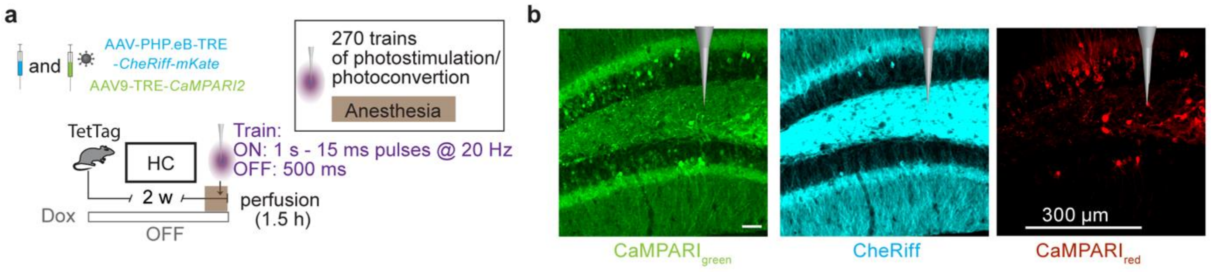

Supplementary Figure 7. Estimating the radius of photoconversion by coexpression of CaMPARI and channelrhodopsin. a Mice were injected a mix of two AAVs encoding CaMPARI2 and CheRiff, respectively. After 2 weeks, mice were anesthetized and stimulated with violet light through a tapered fiber positioned in the hilus, which depolarized the cells and photoconverted calcium-bound CaMPARI. **b** Immunohistochemistry of DG showed photoconverted neurons up to 300 µm away from the fiber tip (red). Based on these calibration experiments, we evaluated neurons within a 200 µm radius around the fiber tip in the behavioral experiments (Fig. 4).

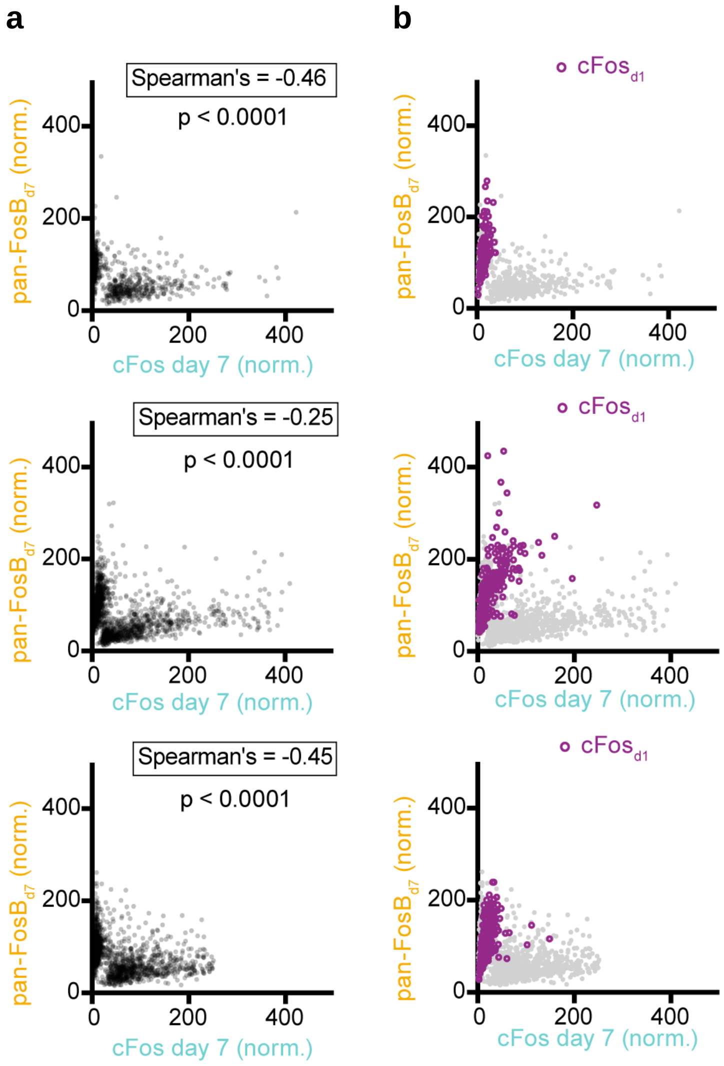

Supplementary Figure 8. Expression analysis in DG in individual mice. a Inverse correlation between cFos expression levels and FosB/ΔFosB on day 7 of WM training. **b** cFos-expressing neurons from day 1 (magenta) typically expressed FosB/ΔFosB, but rarely cFos on day 7.

These data are presented as pooled plots in main Fig. 6b and d.

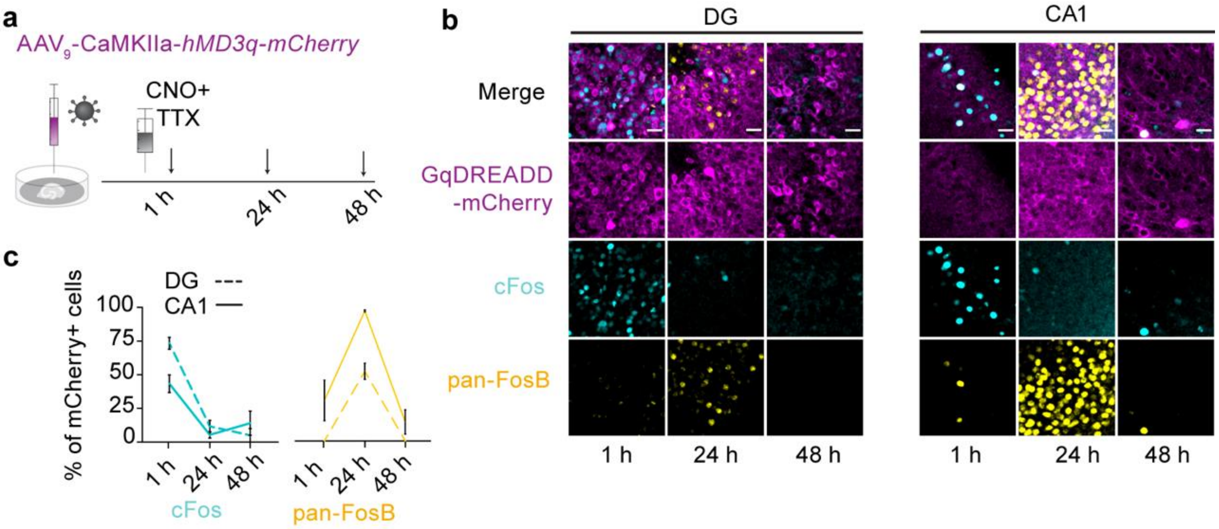

Supplementary Figure 9. Chemogenetic activation of cFos and pan FosB. a Hippocampal slice cultures were transduced with AAV8-CaMKIIa-*GqDREADD-mCherry*. Cultures were silenced with TXX, stimulated with CNO and fixed at 3 different time points. **b** Cultures were immunostained for cFos and pan-FosB. **c** cFos expression was strongly induced by CNO, but rapidly dropped to very low levels after 24 h in both DG and CA1 (mean +/- SEM). Pan-FosB immunoreactivity peaked after 24 h, with a higher fraction of CA1 neurons expressing FosB compared to DG. After 48 h, expression was down in both CA1 and DG.

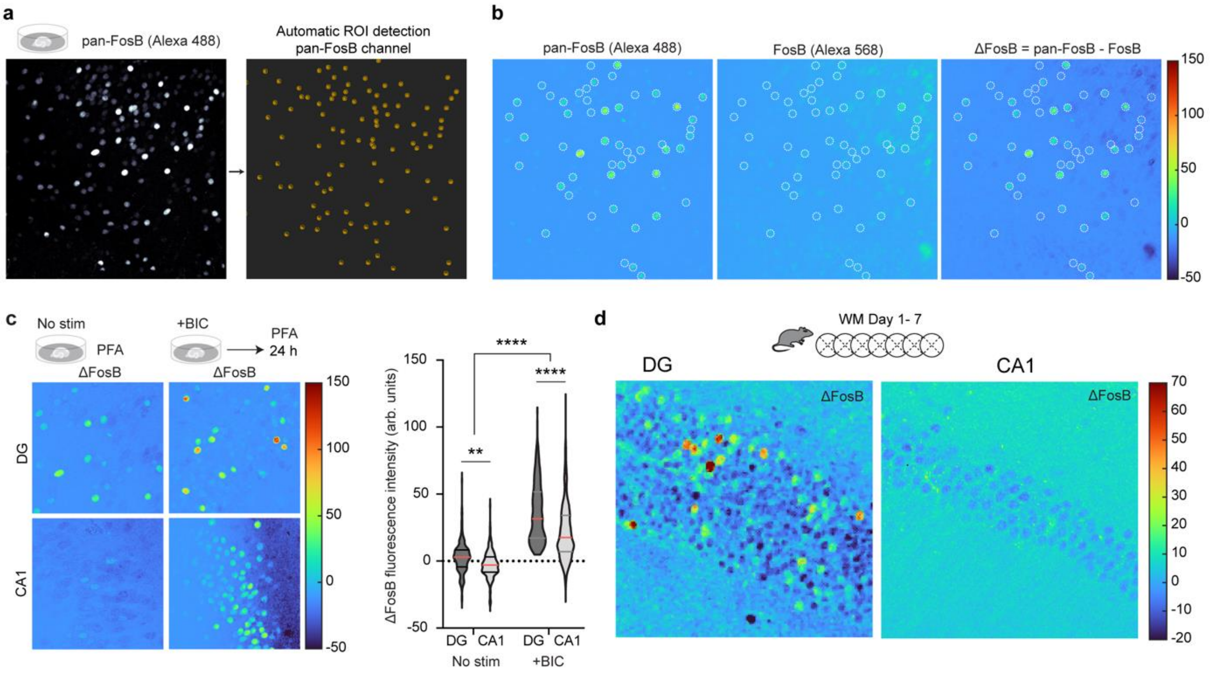

Supplementary Figure 10. Subtraction method to estimate ΔFosB expression. a, Example of an image (3D rendering from stack, Imaris) showing pan-FosB immunoreactive cells in the dentate gyrus (DG) from an organotypic rat slice culture taken at baseline without stimulation. An automated ROI selection was used to place spots (Imaris) on Pan-FosB immunoreactive cells. **b,** Same example image presented with Jet color look-up-table to represent fluorescence intensity of pan-FosB and FosB. Whole image fluorescence subtraction performed in Matlab allowing signed pixel values: pan-FosB channel – FosB channel = ΔFosB. White doted-line circles are placed to indicate the ROIs based on the Pan-FosB immunoreactive cells (placed manually on a single plane so not all spots from **a** appear in this image). **c,** Exemplary single plane images of DG and CA1 of organotypic slice cultures showing ΔFosB levels without stimulation (baseline) and 24 h after bicuculine (BIC) stimulation. Violin plots (range, median ± quartiles): ΔFosB intensities in defined ROIs (Imaris) are significantly higher in DG than in CA1 at baseline (** $p = 0.006$, DG: $n = 228$ cells, 3 slices; CA1: $n = 298$ cells, 3 slices, two-way-ANOVA) and 24 h after BIC stimulation (**** $p < 0.0001$, DG: $n = 435$ cells, 3 slices; CA1: $n = 459$ cells, 3 slices, two-way-ANOVA). **d,** Fluorescence subtraction method applied to brain slices from a mouse that was trained for 7 days in the water maze (WM). Image showing accumulation of ΔFosB in granule cells of the DG but not in pyramidal cells of the CA1 region. Complete statistics in Supplementary Table 1.

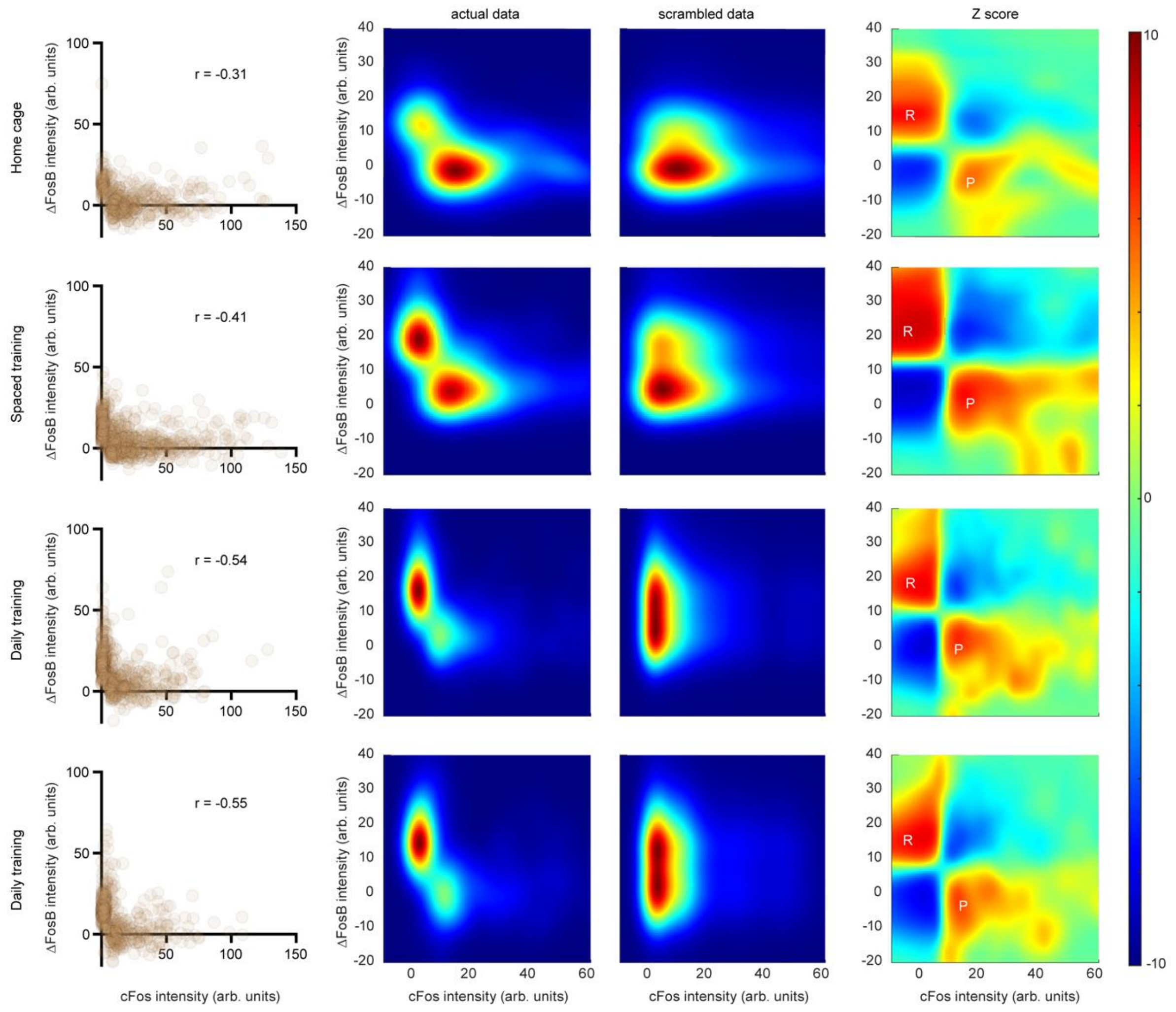

Supplementary Figure 11. Kernel density estimates in DG, more examples. Single cell analysis of cFos and ΔFosB expression in DG after 3 different training regimes. Each row shows data from one mouse (Home cage, spaced training, daily training, daily training). **First column:** Expression of cFos and ΔFosB in individual granule cells (brown circles). Spearman's r indicates inverse correlation. **Second column:** Kernel density estimate from actual single cell data (first column). **Third column:** Kernel density estimate, average of 100 randomized (scrambled) combinations of the actual data. **Fourth column:** Pixel-wise Z-score of actual data compared to scrambled data, scaled from -10 to +10. In every animal, we found two regions of overrepresented cells (positive Z-scores): Low cFos - high ΔFosB (R, repressed) and high cFos - low ΔFosB (P, permissive). In the homecage, most GC are in the permissive state. Spaced training resulted in a bimodal distribution. Daily training shifted the majority of GC to the repressed state.

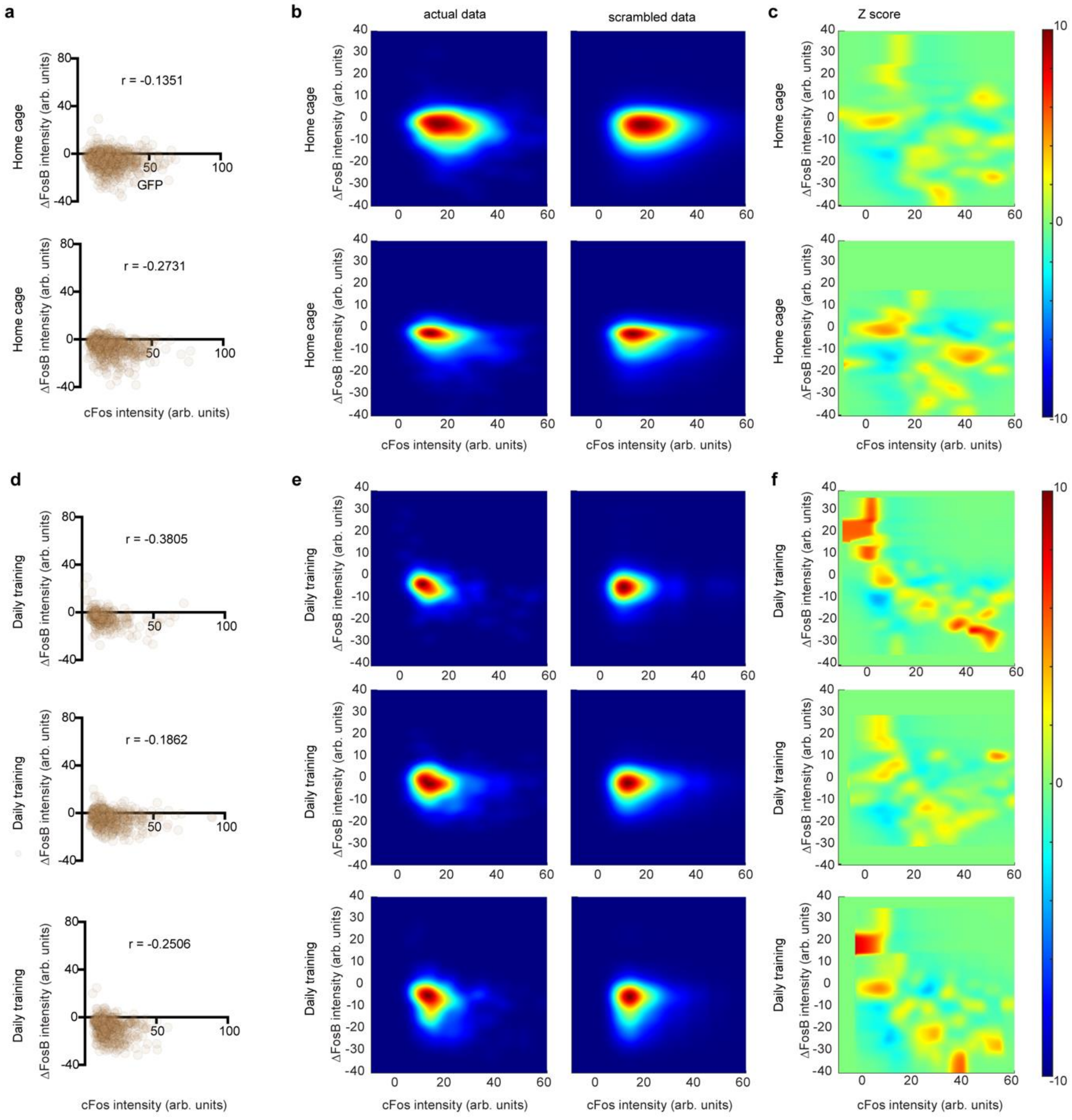

Supplementary Figure 12. Kernel density estimates from CA1 pyramidal cells. a, Two mice were kept in the homecage for 7 days. Expression of cFos and ΔFosB in individual CA1 cells (brown circles). Spearman's r indicates weak inverse correlation. **b,** Kernel density estimate from actual single cell data (first column) and average of 100 randomized (scrambled) combinations of the actual data. **c:** Pixel-wise Z-score of actual data compared to scrambled data, scaled from -10 to +10. **d,** Three mice were trained daily in the water maze for 7 days. Expression of cFos and ΔFosB in individual CA1 cells (brown circles). Spearman's r indicates inverse correlation. **e,** Kernel density estimate from actual single cell data (first column) and average of 100 randomized (scrambled) combinations of the actual data. **f,** Pixel-wise Z-score of actual data compared to scrambled data, scaled from -10 to +10. The actual distributions are similar to the scrambled distributions even after 7 days of WM training.

Supplementary Table 1

Figure	Panel	Groups	n	# replications	Test	Multiple comparison
1	c	ET (days 1-2) + OT (days 1-6)	12 mice (6 per group)	Pooled data from 4 different batches of mice	Mixed model ANOVA. Training (Days) fixed effect: *** p=0.0007	Sidak (Day 1 vs. 2, * p=0.02; day 5 vs. 6 P>0.98)
1	f	ET	6 mice	Pooled data from 4 different batches of mice	Matched, Two-way-ANOVA. Annulus effect: F (1, 5) = 28.68, P=0.0031. Annulus x Tagging Day interaction: F (1, 5) = 4.31, ns p=0.0925	Sidak (Day 1, annulus E vs. W, * p=0.03; Day 2 annulus E vs. W, ** p=0.001
1	h	OT	6 mice	Pooled data from 4 different batches of mice	Matched, Two-way-ANOVA. Annulus effect: F (1, 5) = 36.91, P=0.0017, Annulus x Tagging Day interaciton: F (1, 5) = 1.72, ns p=0.24	Sidak (Day 5, annulus E vs. W, **** p<0.0001; Day 6 annulus E vs. W, ** p<0.0001
1	i	ET and OT	6 mice per group	Pooled data from 4 different batches of mice	Mixed, Two-way-ANOVA. Group: F (1, 10) = 48.77, **** p<0.0001. Group x Tagging Day (learning) interaction: F (1, 10) = 5.28, * p=0.04	Sidak (ET group: day 1 vs. 2, distance to platform E, ** p=0.004; Group OT day 5 vs. 6 distance to platoform E, ns p=0.65.
1	k	ET and OT	6 mice per group	Pooled data from 4 different batches of mice	From SF 3	From SF 3
1 / 2	k / f	ET and OT / RT, NE and HC	6 mice per group	Pooled data from 4 different batches of mice	Ordinary one-way-ANOVA, Group: F (4, 26) = 9.567, **** p<0.0001	Sidak (HC vs. NE: ns p>0.99; HC vs. ET: **p=0.002; HC vs. OT: * p=0.01; ; HC vs. RT: ** p=0.024; NE vs. ET: ** p=0.03; NE vs. OT: * p=0.02; NE vs. RT: ** p=0.29; ET vs. OT: ns p=0.99; ET vs. RT: ns p>0.99; OT vs. RT: ns p=0.98
2	b	RT	6 mice	Pooled data from 4 different batches of mice	Matched, Two-way-ANOVA. Annulus effect: F (1, 5) = 54.68, *** P=0.0007. Annulus x Tagging day interaction: F (1, 5) = 7.49, * p=0.04	Sidak (Day 5, annulus E vs. W, * p=0.01; Day 6 annulus E vs. W, ns p=0.75
2	d	NE	6 mice	Pooled data from 4 different batches of mice	Two-sided paired t test. Annulus E vs. W. ns, p=0.16. CI -3.313 to 15.02	NA
2	f, g	ET, OT, RT, NE, HC	n=1815, 2209, 2524, 1531, 1575 cells	Pooled data from 4 different batches of mice	Ordinary two-way-ANOVA. Group effect: F(4, 40) = 13.19, **** p<0.0001	Sidak (HC vs. ET: **** p<0.0001; HC vs. OT: *** p=0.0003; HC vs. RT: ** p=0.001; ; HC vs. NE: ns p=0.75; ET vs. OT: ns p=0.6 ;ET vs. RT: ns p=0.26 ; ET vs. NE: *** p=0.0002; OT vs. RT: ns p>0.99 ; OT vs. NE: * p=0.03; RT vs. NE: ns p=0.11)
3	b	Optogenetic silencing group	15 mice	Pooled data from 2 different batches of mice	Repated measures one-way-ANOVA. Training (days) effect: F (2.9, 41), **** p<0.0001	Sidak (Escape Latency comparied to first training day. Day 2, *** p=0.0008; day 3, *** p=0.0006, day 4, *** p=0.0002; Day 5, **** p<0.0001
3	c	Tethered vs. Untethered	15 mice	Data from 2 different batches	Two-sided paired t test. Tethered vs. Untethered trials (Day 5) ns p=0.12	NA
3	d	473nm Light vs. No Light	15 mice	Data from 2 different batches	Two-sided paired t test. 473nm Light vs. No Light trials (Day 5) ns p=0.5	NA
3	d	594nm Light vs. No Light	8 mice	1 batch of mice	Two-sided paired t test. 594nm Light vs. No Light trials (Day 5) ns p=0.74	NA
3	g	473nm Light vs. No Light	8 mice (1 mouse excluded)	1 batch of mice	Matched two-way-ANOVA. Quadrant effect: F (3, 18) = 14.91, **** p<0.0001. 473nm Light x Quadrant interaction: F (1, 6) = 0.026, ns p=0.12	Sidak (Time in Quadrant, 473nm Light vs. No Light: South ns p=0.41; East ns p=0.35; North ns p=0.95, West ns p=0.92)
3	h	473nm Light vs. No Light	15 mice (3 mice excluded)	Data from 2 different batches of mice (dotted/solid lines)	Matched two-way-ANOVA. Quadrant effect: F (3, 33) = 68.41, **** p<0.0001. 473nm Light x Quadrant interaction: F (3, 33) = 6.93, *** p=0.001.	Sidak (Time in Quadrant, 473nm Light vs. No Light: South ns p=0.42; East ** p=0.002; North ns p=0.1, West ns p=0.99)
3	i	473nm Light vs. No Light	15 mice (4 mice excluded). Day 2 (n=8), Day 3 (n=7), & Day 5 (n=11)	Data from 2 different batches of mice (dotted/solid lines)	Two-sided paired t test. 473nm Light vs. No Light trials. Day 2: ns p=0.67; Day 3: ** p=0.004; Day 5: ** p=0.002	NA
5	c	Δ5d RT vs. Δ1d RT	Δ5d RT (n= 7 mice) Δ1d RT (n= 6 mice from Fig 2)	Pooled data from 2 different batches of mice	Two-sided unpaired t test. Δ5d RT vs. Δ1d RT overlap/chance. ** p=0.003	NA
5	g	DG: Δ0h, Δ6h,Δ12h, Δ24h	3 mice per group	Pooled data from 3 different batches of mice	Ordinary one-way-ANOVA. Group effect: F (3, 8) = 127, **** p<0.0001	Sidak (Δ0h vs Δ6h ** p=0.005; Δ0h vs. ,Δ12h **** p<0.0001, Δ0h vs., Δ24h **** p<0.0001, , Δ6h vs. Δ12h *** p=0.0005, Δ6h vs. Δ24h **** p<0.0001, Δ12h vs. Δ24h ** p=0.002)

Supplementary Table 1 (cont.)

Figure	Panel	Groups	n	# replications	Test	Multiple comparison
5	g	CA1: Δ0h, Δ6h,Δ12h, Δ24h	3 mice per group	Pooled data from 3 different batches of mice	Ordinary one-way-ANOVA. Group effect: F (3, 8) = 31.24, **** p<0.0001	Sidak (Δ0h vs Δ6h *** p=0.0005; Δ0h vs. ,Δ12h *** p=0.0002, Δ0h vs., Δ24h *** p=0.0003, , Δ6h vs. Δ12h ns p=0.89, Δ6h vs. Δ24h ns p=0.99, Δ12h vs. Δ24h ns p=0.99
6	b	Δ7d OT	6 mice	Data from 1 batch of mice	Spearman r = -0.36, CI: -0.3904 to -0.3358. **** p < 0.0001	NA
6	e	Δ7d OT	6 mice	Data from 1 batch of mice	Two-sided paired t test. Observed vs. Expected Overlap. ** p=0.002	NA
6	f	Δ7d OT	6 mice	Data from 1 batch of mice	Two-sided paired t test. Observed vs. Expected Overlap. *** p=0.0004	NA
7	a upper	NT, Veh., 1st CNO, 2nd CNO	NT (7 slices), Veh. (5 slices), 1st CNO (9 slices), 2nd CNO (14 slices)	Pooled data from 3 replications	Ordinary one-way-ANOVA, Group effect: F(3, 31) = 10.57. **** p<0.0001	Sidak (NT vs. Veh.: ns p>0.99; NT vs. 1st CNO: *** p=0.0003; NT vs. 2nd CNO: ns p=0.88; Veh. vs. 1st CNO: *** p=0.0006; Veh.. vs. 2nd CNO ns p=0.81; 1st CNO vs. 2nd CNO: *** p=0.0008)
7	a lower	NT, Veh., 1st CNO, 2nd CNO	NT (7 slices), Veh. (5 slices), 1st CNO (9 slices), 2nd CNO (14 slices)	Pooled data from 3 replications	Ordinary one-way-ANOVA, Group effect: F(3, 31) = 6.32 ** p=0.0036	Sidak (NT vs. Veh.: ns p>0.99; NT vs. 1st CNO: ns p=0.63; NT vs. 2nd CNO: * p=0.02; Veh.. vs. 1st CNO: ns p=0.77; Veh.. vs. 2nd CNO * p=0.003; 1st CNO vs. 2nd CNO: ns p>0.99)
7	b upper	NT, Veh., 1st CNO, 2nd CNO	NT (7 slices), Veh. (5 slices), 1st CNO (9 slices), 2nd CNO (14 slices)	Pooled data from 3 replications	Ordinary one-way-ANOVA, Group effect: F(3, 31) = 13.70. **** p<0.0001	Sidak (NT vs. Veh.: ns p>0.99; NT vs. 1st CNO: ** p=0.0069; NT vs. 2nd CNO: **** p<0.0001; Veh.. vs. 1st CNO: ** p=0.0067; Veh.. vs. 2nd CNO *** p=0.0002; 1st CNO vs. 2nd CNO: ns p=73)
7	b lower	NT, Veh., 1st CNO, 2nd CNO	NT (7 slices), Veh. (5 slices), 1st CNO (9 slices), 2nd CNO (14 slices)	Pooled data from 3 replications	Ordinary one-way-ANOVA, Group effect: F(3, 31) = 38.52 **** p<0.0001	Sidak (NT vs. Veh.: ns p=0.97; NT vs. 1st CNO: ns p=0.98; NT vs. 2nd CNO: **** p<0.0001; Veh.. vs. 1st CNO: ns p=0.87; Veh.. vs. 2nd CNO **** p<0.0001; 1st CNO vs. 2nd CNO: **** p<0.0001)
8	b	Home cage (HC), Spaced Training (ST), Daily Training (DT)	HC (n = 2), ST (n = 2), DT (n = 3)	Data from 1 batch of mice	Kruskal-Wallis test, **** p < 0.0001	Multiple Dunn's comparison test. HC vs. ST: **** p < 0.0001 ; HC vs. DT: **** p < 0.0001 ; ST vs. DT: ** p = 0.0094.
8	c	HC	1 mouse (example)	replicate in Supp. Fig. 11	Spearman r = -0.14, CI:-0.2284 to -0.05460. ** p = 0.0011	NA
8	c	ST	1 mouse (example)	replicate in Supp. Fig. 11	Spearman r = -0.44, CI: -0.4931 to -0.3944. **** p < 0.0001	NA
8	c	DT	1 mouse (example)	replicate in Supp. Fig. 11	Spearman r = -0.55, CI: -0.6054 to -0.4969. **** p < 0.0001	NA
SF 1	d	cFos and shEGFP	3 slices per time point per group	Data from 1 batch of slices	Matched two-way-ANOVA. Time Point effect: F (4, 16) = 19.74, **** p<0.0001. Time Point x Protein interaction: F (4, 16) = 3.02, * p=0.048	Sidak (cFos expression comparied to baseline (0 min): 30 min ns p=0.69; 60 min **** p<0.0001; 120 min *** p=0.0001; 240 min ** p=0.009) (shEGFP expression comparied to baseline (0 min): 30 min ns p=0.99; 60 min ns p=0.44; 120 min ** p=0.001; 240 min * p=0.02
SF 1	e	0, 30, 60, 120, 240 mins	n=181, 720, 2785, 1759, 1498 cells	Data from 1 batch of slices	Simple linear regression. R^2 = 0.45, 0.09, 0.32, 0.56, 0.57	NA
SF 2	a	On-Dox, HC, NE, ET, OT, RT	On-Dox (n=2), HC (n=7), NE, ET, OT, RT (n=6 per group)	Pooled data from 4 different batches of mice	Ordinary one-way-ANOVA. Group effect: F (5, 25) = 8.04 **** p<0.0001	Sidak (On-Dox vs. HC ** p=0.004, On-Dox vs. NE **** p>0.0001, On-Dox vs. ET ** p=0.001, On-Dox vs. OT *** p=0.0001, On-Dox vs. RT *** p=0.0003,HC vs. NE ns p=0.23, HC vs. ET ns p>0.99, , HC vs. OT ns p=0.49, HC vs. RT ns p=0.84, NE vs. ET ns p=0.64, NE vs. OT ns p>0.99, NE vs. RT ns. p=0.99, ET vs OT ns p=0.9, ET vs RT ns p=0.99, OT vs. RT ns p>0.99)
SF 2	b	HC, NE, ET, OT, RT	HC (n=7), NE, ET, OT, RT (n=6 per group)	Pooled data from 4 different batches of mice	Ordinary one-way-ANOVA. Group effect: F (4, 26) = 3.41 * p=0.027	Sidak ,HC vs. NE ns p=0.52, HC vs. ET ns p>0.26, , HC vs. OT * p=0.02, HC vs. RT * p=0.04, NE vs. ET ns p=0.98, NE vs. OT ns p=0.5, NE vs. RT ns. p=0.65, ET vs OT ns p=0.78, ET vs RT ns p=0.89, OT vs. RT ns p=0.99)
SF 2	c	ET, OT, RT, NE, HC, Δ5d RT	6 mice per group, except HC (7)	Pooled data from 4 different batches.	Matched, Two-way-ANOVA.Group effect: F (5, 32) = 7.21, *** p=0.0001. Group x Observed vs. Expected interaction F (4, 26) = 7.63, *** p=0.0003	Sidak (Observed vs. expected (chance), HC group: ns p=0.99; NE group: ns p=0.74; ET group: *** p=0.0008; OT group: ** p=0.003; RT group: *** p=0.0002; Δ5d RT: ns p=0.52

Supplementary Table 1 (cont.)

Figure	Panel	Groups	n	# replications	Test	Multiple comparison
SF 2	d	DG: Δ0h, Δ6h,Δ12h, Δ24h	3 mice per group	Pooled data from 4 different batches.	Ordinary one-way-ANOVA. Group effect: $F (3, 8) = 0.53$ ns $p=0.67$	Sidak (Δ0h vs Δ6h ns p=0.99; Δ0h vs. ,Δ12h ns p=0.99, Δ0h vs., Δ24h ns p=0.96, , Δ6h vs. Δ12h ns p=98, Δ6h vs. Δ24h ns p=0.99, Δ12h vs. Δ24h ns p=0.82
SF 2	d	CA1: Δ0h, Δ6h,Δ12h, Δ24h	3 mice per group	Pooled data from 4 different batches.	Ordinary one-way-ANOVA. Group effect: $F (3, 8) = 3.25$ ns $p=0.08$	Sidak (Δ0h vs Δ6h ns p=0.37; Δ0h vs. ,Δ12h ns p=0.96, Δ0h vs., Δ24h ns p=0.58, , Δ6h vs. Δ12h ns p=2, Δ6h vs. Δ24h ns p=0.06, Δ12h vs. Δ24h ns p=0.83
SF 5	c	HC vs HC + Optogenetic Reactivation	HC (n=7), HC + Opto ractivation (n=3)	Data from 1 batch of mice	Two-sided unpaired t test. HC overlap vs. HC overlap + Optogenetic Reactivation: *** p=0.003	NA
SF 5	e	594nm Light vs. No Light	8 mice	Data from 1 batch of mice	Matched two-way-ANOVA. Quadrant effect: $F (3, 21) = 37.73$, **** $p<0.0001$. 594nm Light x Quadrant interaction: $F (1, 7) = 0.04$, ns $p=0.98$	Sidak (Time in Quadrant, 595nm Light vs. No Light: South ns p=0.99; East ns p>0.99; North ns p=0.99, West ns p>0.99)
SF 5	f	594nm Light vs. No Light	8 mice	Data from 1 batch of mice	Two-sided paired t test. Time in Annulus E (594 nm light vs. No Light): ns p=0.49	NA
SF 6	b	WM Chemogenetic Silencing	10 mice	Data from 1 batch of mice	Simple linear regression. R square 0.67, ** p=0.003.	NA
SF 6	d	WM Chemogenetic Silencing: Low Expression & High Expression	Low Expression 6 mice, High Expression 4 mice	Data from 1 batch of mice	Two-sided unpaired t test. Low vs. High GiDREADD-mCherry Expression: * p=0.01	NA
SF 6	d	WM Chemogenetic Silencing: Low Expression & High Expression	Low Expression 6 mice, High Expression 4 mice	Data from 1 batch of mice	Two-sided unpaired t test. Low vs. High GiDREADD-mCherry Expression: ** p=0.007	NA
SF 6	e	WM Chemogenetic Silencing: High Expression Group	4 mice	Data from 1 batch of mice	Two-sided paired t test. Vehicle vs. CNO: ns p=0.07	NA
SF 6	f	WM Chemogenetic Silencing: High Expression Group	4 mice	Data from 1 batch of mice	Two-sided paired t test. day 7 (Vehicle) vs. day 8(CNO): ns p=0.53	NA
SF 6	h	Subset of mice from the WM Chemogenetic Silencing Group to test CNO effect on cFos expression	3 slices from 1 mouse	Data from 1 batch of mice	Two-sided paired t test. Inside vs. Outside of mCherry+ regions: ns p=0.07	NA
SF 8	a	Δ7d OT	1 mouse (example 1)	3	Spearman r = -0.46, CI: -0.5168 to -0.3938. **** p < 0.0001	NA
SF 8	a	Δ7d OT	1 mouse (example 2)		Spearman r = -0.25, CI: -0.2982 to -0.2075. **** p < 0.0001	NA
SF 8	a	Δ7d OT	1 mouse (example 3)		Spearman r = -0.45, CI: -0.4923 to -0.4146. **** p < 0.0001	NA
SF 10	c	No stimulation, Bicucculine (BIC)	3 slices per group		Unmatched, Two-way-ANOVA $F (1, 1416)$. Region effect: **** p=0.0001. Stimulation effect **** p=0.0001. Region X Stimulation interaction **** p=0.0001	Sidak (DG-noStim. vs. DG-BIC: **** p < 0.0001; DG-noStim. vs. CA1-noStim.: ** p = 0.006; DG-noStim. vs. CA1-BIC: **** p < 0.0001; DG-BIC vs. CA1-noStim: **** p < 0.0001; CA1-noStim vs. CA1-BIC: **** p < 0.0001
SF 11	NA	HC	1 mouse (example)	replicate in Fig. 8	Spearman r = -0.31, CI: -0.3921 to -0.2197. **** p < 0.0001	NA
SF 11	NA	ST	1 mouse (example)	replicate in Fig. 8	Spearman r = -0.41, CI: -0.4661 to -0.3470. **** p < 0.0001	NA
SF 11	NA	DT	1 mouse (example)	replicate in Fig. 8	Spearman r = -0.54, CI: -0.5944 to -0.4766. **** p < 0.0001	NA
SF 11	NA	DT	1 mouse (example)	replicate in Fig. 8	Spearman r = -0.55, CI: -0.6124 to -0.4814. ** p = 0.0011	NA
SF 12	c	HC	1 mouse (example)		Spearman r = -0.14, CI: -0.2122 to -0.05640. *** p = 0.0006	NA
SF 12	c	HC	1 mouse (example)		Spearman r = -0.27, CI: -0.3477 to -0.1950. **** p < 0.0001	NA
SF 12	c	DT	1 mouse (example)		Spearman r = -0.38, CI: -0.4778 to -0.2739. **** p < 0.0001	NA
SF 12	c	DT	1 mouse (example)		Spearman r = -0.19, CI: -0.2732 to -0.09618. **** p < 0.0001	NA
SF 12	c	DT	1 mouse (example)		Spearman r = -0.25, CI: -0.3341 to -0.1631. **** p < 0.0001	NA

Supplementary Table 2

Primary and secondary antibodies

Immunogen	Host	Producer	Factory#	Dilution
cFos	Rat	Synaptic System	226017	1:1000
FosB/ΔFosB	Rabbit	Cell Signaling	#2251	1:1000
tRFP (mKate2)	Rabbit	Evrogen	AB233-EV	1:1000
GFP	Chicken	Invitrogen	A10262	1:1000
Anti-Campari2 red (4F61)	Rabbit	Absolute Antibody	AB1649-23.0	1:1000
FosB	Rabbit	Thermofisher	PA5-79280	1:300
FosB/ΔFosB	Mouse	Abcam	Ab11959	1:300

Immunogen/label	Host	Producer	Factory#	Dilution
Rabbit-488	Goat	Invitrogen	A11008	1:1000
Rabbit-568	Goat	Invitrogen	A11011	1:1000
Chicken-488	Goat	Invitrogen	A11039	1:1000
Rabbit-647	Goat	Invitrogen	A27040	1:1000
Rat-647	Goat	Invitrogen	A21247	1:1000
Mouse-488	Goat	Invitrogen	A11029	1:1000
Mouse-405	Goat	Invitrogen	A31553	1:1000
