# Supplementary material for: ΔFosB accumulation in hippocampal granule cells drives cFos pattern separation during spatial learning
Source: Nat Commun. 2022 Oct 26;13:6376. doi: 10.1038/s41467-022-33947-w (PMC9606265; doi:10.1038/s41467-022-33947-w)
Supplement: Supplementary file 3 — Description of Additional Supplementary Files [file 41467_2022_33947_MOESM3_ESM.pdf]

Description of Additional Supplementary Files

Supplementary Movie 1: Optogenetic silencing of cFos-tagged granule cells from the dentate gyrus impairs spatial memory recall. Exemplary video of an expert mouse during two consecutive spatial memory tests (probe trials) in the water maze (WM). No blue (473 nm) light is delivered during the first probe trial and the mouse spends most of the time in the target quadrant (blue shade). When blue light is delivered, the percentage of time the mouse spends in the target quadrant is significantly decreased. Blue light effect is due to the optogenetic inhibition of granule cells from the dentate gyrus that expressed cFos on the first day of WM training (cFostagged). Supplementary to Fig. 3.
