# Supplementary material for: ΔFosB accumulation in hippocampal granule cells drives cFos pattern separation during spatial learning
Source: Nat Commun. 2022 Oct 26;13:6376. doi: 10.1038/s41467-022-33947-w (PMC9606265; doi:10.1038/s41467-022-33947-w)
Supplement: Supplementary file 5 — Reporting Summary [file 41467_2022_33947_MOESM5_ESM.pdf]

nature portfolio

Corresponding author(s): Thomas G. Oertner, Paul J. Lamothe Molina

Last updated by author(s): Sep 29, 2022

Reporting Summary

Nature Portfolio wishes to improve the reproducibility of the work that we publish. This form provides structure for consistency and transparency in reporting. For further information on Nature Portfolio policies, see our Editorial Policies and the Editorial Policy Checklist.

Statistics

For all statistical analyses, confirm that the following items are present in the figure legend, table legend, main text, or Methods section.

n/a	Confirmed	
☐	☒	The exact sample size (n) for each experimental group/condition, given as a discrete number and unit of measurement
☐	☒	A statement on whether measurements were taken from distinct samples or whether the same sample was measured repeatedly
☐	☒	The statistical test(s) used AND whether they are one- or two-sided<br>*Only common tests should be described solely by name; describe more complex techniques in the Methods section.*
☒	☐	A description of all covariates tested
☐	☒	A description of any assumptions or corrections, such as tests of normality and adjustment for multiple comparisons
☐	☒	A full description of the statistical parameters including central tendency (e.g. means) or other basic estimates (e.g. regression coefficient) AND variation (e.g. standard deviation) or associated estimates of uncertainty (e.g. confidence intervals)
☐	☒	For null hypothesis testing, the test statistic (e.g. F, t, r) with confidence intervals, effect sizes, degrees of freedom and P value noted<br>*Give P values as exact values whenever suitable.*
☒	☐	For Bayesian analysis, information on the choice of priors and Markov chain Monte Carlo settings
☒	☐	For hierarchical and complex designs, identification of the appropriate level for tests and full reporting of outcomes
☒	☐	Estimates of effect sizes (e.g. Cohen's d, Pearson's r), indicating how they were calculated

Our web collection on statistics for biologists contains articles on many of the points above.

Software and code

Policy information about availability of computer code

Data collection	Ethovision XT 11.5 for mouse tracking and behavioral analysis. FluoView FV 1000 for confocal image acquisition (Olympus). Zen 3.5 for confocal image acquisition (Zeiss).
Data analysis	Imaris 9.7 (Bitplane) for cell count and fluorescence intensity analysis. ImageJ 1.53 (Fiji) for pixel-wise image division. Numerical data from individual experiments were collected and ensemble sizes calculated in MS Excel (version 16.0). GraphPad Prism 8 for data plotting and statistical analysis. Custom code to generate kernel density estimates in Matlab and to simulate cFos suppression has been deposited at GitHub (https://github.com/toertner/Kernel-density-estimate).

For manuscripts utilizing custom algorithms or software that are central to the research but not yet described in published literature, software must be made available to editors and reviewers. We strongly encourage code deposition in a community repository (e.g. GitHub). See the Nature Portfolio guidelines for submitting code & software for further information.

Data

Policy information about availability of data

All manuscripts must include a data availability statement. This statement should provide the following information, where applicable:

- Accession codes, unique identifiers, or web links for publicly available datasets
- A description of any restrictions on data availability
- For clinical datasets or third party data, please ensure that the statement adheres to our policy

Numerical data of all figures is provided (Source_Data_Lamothe_2022.xlsx).

Field-specific reporting

Please select the one below that is the best fit for your research. If you are not sure, read the appropriate sections before making your selection.

- [x] Life sciences
- [ ] Behavioural & social sciences
- [ ] Ecological, evolutionary & environmental sciences

For a reference copy of the document with all sections, see nature.com/documents/nr-reporting-summary-flat.pdf

Life sciences study design

All studies must disclose on these points even when the disclosure is negative.

Sample size	Sample size was set according to published studies using similar experimental designs (see references). Final sample size was set to 15 mice per group for optogenetic manipulation of behavior. To investigate cFos overlap at different stages of learning, we used 6 mice per group and analyzed 6 brain slices per animal. CaMPARI photoconversion during behavior was performed in 2 mice (no statistical analysis). For full statistical information, please consult Supplementary Table 1.
Data exclusions	Mice that showed no transgene expression were excluded from analysis. Mice with off-target implant site or very slow swim speed were not included in the analysis (numbers provided in Supplementary Table 1). Two batches of immunostainings had (for unknown reasons) very low fluorescence intensities and were excluded from the expression level analysis in Fig. 2g.
Replication	In our study, main sources of variability were 1) stereotactic virus injection (location, injected volume, titer), 2) Fiber implantation (tip position). 2) motivation / stress level in behavioral experiments, 3) transcardial perfusion and antibody staining procedure (immunoreactivity). To minimize systematic errors, we performed the water maze experiments in batches of animals that were trained, tested, killed and stained together. Whenever possible, mice from one batch were assigned to several different experimental groups, e.g. trained and control (home cage) animals. As we replicated the same experimental treatments and immunohistochemical measurements across several batches of animals (several weeks apart), we expect the results are robust and reproducible. Information about the number of batches in each experiment is provided in Supplemental Table 1.
Randomization	Mice were similar in age and littermates. After surgery, mice were assigned into experimental groups by randomizing the animal ID number to a group. Except in optogenetic experiments, both sexes were included and counterbalanced. In experiments where a group was further subdivided into two (e.g. Fig. 3), we performed statistical tests to compare behavioral parameters to ensure there was no selection bias.
Blinding	All histological image acquisition and analysis were performed blind to experimental conditions. All behavioral analysis was performed by an automated software. Some swimming tracks contained artifacts (ballon before camera) that were corrected by an experimenter who was blind to the experimental conditions.

Reporting for specific materials, systems and methods

We require information from authors about some types of materials, experimental systems and methods used in many studies. Here, indicate whether each material, system or method listed is relevant to your study. If you are not sure if a list item applies to your research, read the appropriate section before selecting a response.

Materials & experimental systems

n/a	Involved in the study	
☐	☒	Antibodies
☒	☐	Eukaryotic cell lines
☒	☐	Palaeontology and archaeology
☐	☒	Animals and other organisms
☒	☐	Human research participants
☒	☐	Clinical data
☒	☐	Dual use research of concern

Methods

n/a	Involved in the study	
☒	☐	ChIP-seq
☒	☐	Flow cytometry
☒	☐	MRI-based neuroimaging

Antibodies

Antibodies used	Primary antibodies: Chicken Anti GFP polyclonal antibody, Invitrogen (A10262, Lot 1972783); Rabbit Anti-tRFP (mKate2) antibody, Evrogen (AB233, Lot 23301040466); Rat anti cFos, Synaptic Systems (226 017, Lot 1-6); Rabbit anti FosB (5G4), Cell Signaling Technology (#2251, Lot 3); Rabbit anti FosB, Thermofisher (PA5-79280); Mouse anti FosB, Abcam (Ab11959). Anti-Campari2 red (4F61), Absolute Antibody (AB1649-23.0).<br>Secondary antibodies (Invitrogen): Rabbit-488, A11008; Rabbit-568, A11011; Chicken-488, A11039; Rabbit-647, A27040; Rat-647, A21247; Mouse-488, A11029; Mouse-405, A31553.
Validation	GFP and mKate antibodies were not reactive in non-AAV injected wild-type mice that do not express shEGFP or mKate2. cFos and FosB antibodies did not show unspecific reactivity outside the nucleus. Anti-Campari2 red did not bind to non-photoconverted CaMPARI.

Animals and other organisms

Policy information about studies involving animals; ARRIVE guidelines recommended for reporting animal research

Laboratory animals	B6.Cg-Tg(Fos-tTA,Fos-EGFP*)1Mmay/J (TetTag) mice were obtained from the Jackson Laboratory (Strain #018306) and bred to wildtype (noncarrier) C57BL6/J mice from our colony. Mice were group-housed with littermates until 2 weeks before rAAV injections, then were single-caged. Mice had access to food and water ad libitum and were kept in an animal facility next to the behavioral rooms on a reversed light-dark cycle (dark 7 am - 7 pm) at 20-23°C with 45-65% humidity. All behavioral experiments were done during the dark phase of the cycle. Due to the requirement to swim with optical fibers, only male mice between 20-40 weeks (>28 g by the time of surgery) were used for optogenetic WM experiments (Fig. 3-5). Both male and female mice were included in the cFos ensemble overlap experiments (Fig. 1, 2 & 6).
Wild animals	No wild animals were collected for this study.
Field-collected samples	No field samples were collected for this study.
Ethics oversight	All experiments were conducted in accordance with the German and European Union laws on protection of experimental animals and approved by the local authorities of the City of Hamburg (Behörde für Justiz und Verbraucherschutz (BJV)-Lebensmittelsicherheit und Veterinärwesen- Nr 100/15 and Nr 046/2021

Note that full information on the approval of the study protocol must also be provided in the manuscript.
